# Executive Function and Mental Health in Adopted Children with a History of Recreational Drug Exposures

**DOI:** 10.1371/journal.pone.0110459

**Published:** 2014-10-22

**Authors:** Brian J. Piper, Hilary M. Gray, Selena M. Corbett, Melissa A. Birkett, Jacob Raber

**Affiliations:** 1 Department of Basic Pharmaceutical Sciences, Husson University, Bangor, Maine, United States of America; 2 Department of Behavioral Neuroscience, Oregon Health and Science University, Portland, Oregon, United States of America; 3 School of Community Health and Department of Counselor Education, Portland State University, Portland, Oregon, United States of America; 4 College of Osteopathic Medicine of the Pacific Northwest, Western University of Health Sciences, Pomona, California, United States of America; 5 Department of Psychological Sciences, Northern Arizona University, Flagstaff, Arizona, United States of America; 6 Department of Neurology and Radiation Medicine, and Division of Neuroscience, ONPRC, Oregon Health and Science University, Portland, Oregon, United States of America; Chiba University Center for Forensic Mental Health, Japan

## Abstract

Adoptive children are at increased risk for problematic behaviors but the origin of these individual differences in neurobehavioral function is unclear. This investigation examined whether adopted children with prenatal exposure to a wide variety of recreational drugs exhibited higher scores (i.e. more problems) with executive function and psychiatric symptomology. Caregivers of children ages 5 to 18 completed an online survey with items about use of alcohol, nicotine, or methamphetamine during pregnancy followed by the Behavior Rating Inventory of Executive Function (BRIEF, N = 437 including 59 adoptive parents) or the Child Behavior Checklist (CBCL, N = 549 including 54 adoptive parents). Relative to a comparison group of children raised by their biological parents, adoptive children that were polysubstance exposed during prenatal development exhibited higher rates of academic difficulties and were behind their classmates in math and reading. Adoptive children had statistically and clinically significant higher BRIEF ratings and this pattern was similar for boys and girls. CBCL ratings were significantly increased in adoptive children, particularly for Externalizing and Attention problems. Adoptive children with a history of polysubstance exposures including alcohol, nicotine, and methamphetamine are at heightened risk for difficulties with executive function as well as various psychopathologies. These findings suggest that increased monitoring to identify and implement remediation strategies may be warranted for adopted children with a history of *in utero* drug exposures.

## Introduction

An extensive and long-standing literature has thoroughly documented that adoptive children are over-represented in clinical settings and have academic difficulties [1]. Meta-analyses of parental reports using instruments like the Child Behavior Checklist (CBCL) have documented that domestic adoptees had more total problems, Externalizing problems, and Internalizing problems than international adoptees [2]. The origins and processes responsible for these individual differences has become an area of increasing interest with an emphasis on psychological, endocrine, and genetic factors [1]. The teratogenic potential of prenatal exposure to recreational drugs to contribute to adverse outcomes [3] has received less systematic attention among offspring that are subsequently put-up for adoption. This oversight may be due to a variety of factors including that exact information regarding the timing and extent of drug use may be impractical to obtain from the birth mother [4]. Alternatively, among polysubstance abusing women who may have multiple legal problems who subsequently become involved with child welfare agencies, it may be challenging to isolate the unique contribution of an individual agent from other comorbid conditions including under-utilization of medical services, poor nutrition/decreased rates of breast feeding, maternal stress, domestic violence, trauma, age of the child at adoption, or a sub-optimal socio-economic environment. However, there are at least three reasons why adopted children are an important population to study. First, a mother whose drug use patterns are extensive enough to contribute to the loss of her legal rights to child custody may provide important insights into the risks associated with a particular drug that may not be as readily apparent among the offspring of women with less intense use patterns. Second, adoptive children provide an important natural experiment, analogous to standard practices by preclinical investigations, to begin to untangle the importance of the prenatal and postnatal environment. Third, we have found that adoptive parents, as a group, are strong advocates for their children and valuable participants in research studies which could form the empirical foundation for remediation efforts. Together, these factors form the impetus for this report.

Executive function refers to neurocognitive processes responsible for generating and regulating behavior which includes selective attention, forming plans, working memory, solving problems, and mental flexibility. Executive function elements show a dose-dependent sensitivity to *in utero* exposure to a wide variety of recreational drugs, most prominently alcohol [5], [6] but also nicotine [7], [8], and methamphetamine [9], [10]. Interestingly, girls, but not boys, prenatally exposed to cocaine showed more problems with executive function as determined by a parent completed questionnaire, the Behavior Rating Inventory of Executive Function (BRIEF) [11]. As executive function is an important capacity that also contributes to a wide variety of psychiatric conditions (e.g. Bipolar Disorder), this paper reports on two complementary investigations describing parentally rated behavior in adopted and non-adopted offspring with the BRIEF (Study I) and CBCL (Study II). We hypothesized that there are pronounced abnormalities in executive function and psychopathology, largely independent of child age and sex, among adoptive children with a history of prenatal drug exposures.

## Materials and Methods

Caregivers of children ages 5 to 18 (Study I, N = 437) or 6 to 18 (Study II, N = 539) were recruited for a child behavior investigation which was displayed on the volunteer and community sections of Craigslist (craigslist.org) as well as the Oregon Post-Adoption Resource Center website (orparc.org) and newsletter. Paper flyers were prominently and frequently posted on community boards throughout Oregon Health Science University (OHSU), the Portland metro area, and western Oregon/western Washington (e.g. laundromats, libraries). Participants were not offered an incentive for their participation. This anonymous online survey was administered by Research Electronic Data Capture (REDCap), version 1.3.9, an application for procuring online databases with maximal security for sensitive information [12]. Exclusion criteria were incomplete/unfinished questionnaires and a child age that was outside the range of the instruments (5 to 18 for the BRIEF, 6 to 18 for the CBCL). The Institutional Review Board of OHSU (Study I & II, protocol #5720) as well as Northern Arizona University (Study II, protocol #11.0169) approved all of the procedures including the consent form.

### Measures

After completing an online consent, the caregivers began the survey which typically took about twenty minutes. The first half of the items were organized from less to more sensitive and included questions about maternal and child demographics (e.g. age, sex, ethnicity), academic performance (e.g. “Please rate your child’s performance in reading with relation to their scores on the state’s standardized test.” with options of below, at, or above grade level), and child psychiatric and neurological conditions (e.g. diagnosis of Attention Deficit Hyperactivity Disorder). An additional item was added for study II (What percent of your child’s life have they lived with you?). Questions on maternal drug use were organized into two periods: during pregnancy and specifically during the third trimester. Because pre-adoption histories may not be known with certainty in all cases, the response options for the drugs most likely to be used during pregnancy (alcohol and nicotine) were, yes, no, suspect, or don’t know. The Behavior Rating Inventory of Executive Function (BRIEF) accounted for the remaining 86 items in Study I and the Child Behavior Checklist (CBCL) accounted for the final 118 items in Study II. As data collection was anonymous, there is no simple method to determine if any parents from Study I also participated in Study II.

The BRIEF is a rating instrument completed by parents for the neuropsychological assessment of children and adolescents which focuses on the child’s everyday activities at home and at school. Each behavior is rated as never, sometimes, or often a problem (1 to 3 points, respectively) in the last six months. The eight BRIEF scales form two measures of executive functioning (Metacognition and Behavioral Regulation) and these are totaled for an overall measure (the Global Executive Composite or GEC). The Metacognition Index consists of the following five scales: 1) Working Memory, the capacity to hold information to complete a task; 2) Monitor, self-monitoring habits; 3) Organization of Materials, the extent of orderliness of play and work areas; 4) Plan/Organize, the capability to foresee future events, construct goals, and implement the appropriate steps to complete a task; and: 5) Initiate, the ability to act independently to produce ideas, responses, or problem solving strategies. The Behavioral Regulation Index is composed of three scales: 1) Emotional Control, the ability to regulate emotions appropriately; 2) Shift, the capability to change from one activity to another; and 3) Inhibit, the capacity to regulate one’s behavior at the appropriate time and not act on impulse. Negativity scale scores were obtained by summing the number of select items (maximum = 9) with an “often” response. An inconsistency scale score was determined by calculating the difference between ten item pairs (range = 0 to 20) with a score ≥9 interpreted as inconsistent. Standardized (T_50_ scores) were calculated based on age/sex norms with higher scores indicating greater severity. A small (4.7%) portion of the BRIEF standardization sample (N = 1,417) consisted of grandparents and adoptive/foster parents. The BRIEF has excellent internal consistency (Cronbach’s alpha = 0.80 to 0.98) and very good test-retest reliability (r>0.70) [13], [14]. Additional information about the psychometric properties of the BRIEF including about the moderately large (N = 1,419) normative sample and the traumatic brain injury validation sample is available elsewhere [13], [14].

The CBCL 6–18 parent form measures a child’s problems and can be completed in approximately 20 minutes [16], [17]. Items about the child’s behavior are rated by caregiver as being 0 = not true (as far as you know); 1 = somewhat or sometimes true; or 2 = very true or often true. There are separate scoring profiles based on age (6 to 11 or 12 to 18) and sex. The Syndrome scales are Anxious/Depressed, Withdrawn/Depressed, Somatic Complaints, Social Problems, Thought Problems, Attention Problems (including both Inattention and Hyperactivity-Impulsivity items), Rule-Breaking Behavior, Aggressive Behavior, and Other Problems. The Internalizing broadband scale is the sum of Anxious/Depressed, Withdrawn/Depressed and Somatic Complaints scores. Similarly, the Externalizing broadband scale is the sum of the Rule-Breaking Behavior, and Aggressive Behavior scores. Additional information about the Syndrome scales may be found elsewhere [16]. The CBCL has very good internal consistency (Cronbach’s alpha for total problems = .97) and one-week test-retest reliability (r = .94) whereas cross-informant correlations are appreciably lower [16]. The full survey, excluding copyrighted materials, is available in the Materials S1.

### Data analyses

Statistical analyses were completed with the Systat (Chicago, IL), version 13.0, with data expressed as mean ±SD for tables with ±SEM for figures, and *p*<.05 considered statistically significant. Foster parents were relatively infrequently encountered (N = 3 in Study I and N = 8 in Study II) so were not included in this report. Respondents were divided into two groups, Adoptive (N = 59) and a Comparison group (N = 378) composed of biological mothers (N = 366) or biological fathers (N = 12) for Study I. Similarly, Study II consisted of an Adoptive (N = 54) group and a Comparison group (N = 485) with biological mothers (N = 469) and biological fathers (N = 26). Please note that demographic information and child ratings from the biological mothers is reported elsewhere [8], [15]. Unfortunately, given the use pattern of substances reported, as well as the occurrence of some children where the maternal history was unknown, made it impossible to create a subgroup of adopted children that were unexposed to alcohol, nicotine, or methamphetamine during pregnancy. Analyses were completed on the BRIEF standardized scores and on the percentage of children with clinically significant (T_50_≥65) problems in that domain. CBCL analyses were conducted using the total (raw) scores and using the percentage meeting clinically significant thresholds (i.e. age and sex corrected). Additional analyses were completed for children (age<13) and adolescents (age≥13) separately. Categorical level analyses were completed with a chi-square, or Likelihood ratios if the N/cell was <5. The Odds Ratio (OR) was listed only for significant associations among dichotomous variables. The data for Study I and Study II are available as Materials S1. Key findings were expressed in terms of effect size (Cohen’s *d*) with values of ≈0.20, ≈0.50, or ≥0.80 interpreted as small, medium, or large, respectively.

## Results

### Sample characteristics in Study I

The majority of respondents were from Oregon (48.9%) or Washington (15.2%). Approximately two-thirds (65.8%) of the Comparison group were recruited from Craigslist relative to only one-third (32.2%) of Adoptive parents (χ^2^(1) = 24.4, *p*<.001). Table 1 shows that the Adopted (N = 59) and Comparison (N = 378) groups did not differ significantly in terms of child sex or likelihood of the child being born premature. Adopted children were more likely (OR = 2.8) to be non-white (15.3% Black, 13.6% Alaska Native). Children that were Adopted were significantly more commonly diagnosed with many conditions including Fetal Alcohol Syndrome, a Cognitive Delay (OR = 13.7), Post-Traumatic Stress Disorder (OR = 13.4), Attachment Disorder (OR = 8.6), Motor Development Disorder (OR = 7.1), a Hearing Impairment (OR = 6.8), a birth defect (OR = 6.6), Sensory Integration Disorder (OR = 5.2), a Developmental Disorder (OR = 3.9), Speech Delay (OR = 2.8), or ADHD (OR = 2.2).

**Table 1:** Child and maternal characteristics among respondents completing study I (BRIEF: Behavioral Rating Inventory of Executive Function) or II (CBCL: Child Behavior Checklist).

	Study I: BRIEF				Study II: CBCL			
Child	Adoptive		Comparison		Adoptive		Comparison	
Sex (% female, N)	44.1	59	50.2	378	57.4	54	49.0	492
Age (Mean, SD, N)	9.2, 3.9*	59	10.5, 4.0	378	11.0, 3.7	54	11.5, 3.8	495
Ethnicity(% non-white, N)	35.6**	59	16.6	378	31.5	54	23.9	493
Born premature (%, N)	11.3	52	15.4	377	26.5	43	15.6	483
ADHD (%, N)	35.6*	59	20.4	378	31.5**	54	14.9	495
Fetal AlcoholSyndrome (%, N)	10.2***	59	0.0	378	11.1***	54	0.4	495
School performance(% below, N)	31.0***	58	8.0	376	32.0**	53	13.6	494
Math (% below, N)	44.7**	47	26.6	352	38.8**	49	18.8	462
Reading (% below, N)	42.9***	49	18.3	349	36.0**	50	18.3	465
Family								
Age when BM pregnant (Mean, SD, N)	23.4, 6.1*	53	25.6, 6.5	366	24.8, 7.4	52	25.8, 7.0	492
Education of BM(% < High-school, N)	97.9***	47	29.2	359	95.2***	42	26.4	493
Income of BM(% <$10 K, N)	80.0***	25^D^	17.6	369	88.9***	27^D^	21.1	468
Family income(%, >$50 K, N)	53.8	52	47.7	369	64.8	54	43.5	464
Prenatal Drug Exposure								
Nicotine (% exposed, N)	88.9***	36^SD^	23.6	369^SD^	61.9***	21^SD^	20.	489^SD^
Alcohol (% exposed, N)	87.5***	24^SD^	18.7	374	80.0***	15^SD^	13.9	489^SD^
Methamphetamine(% exposed, N)	71.1***	45^D^	4.2	378	74.1***	27^D^	4.3	492
Marijuana(% exposed, N)	46.7***	45^D^	11.4	378	25.9*	27^D^	10.6	492
Cocaine(% exposed, N)	15.6**	45^D^	3.2	378	22.2***	27^D^	1.8	492
Barbiturates(% exposed, N)	4.4*	45^D^	0.	378	11.1***	27^D^	0.2	492
Oxycontin(% exposed, N)	2.2	45^D^	0.0	378	7.4**	27^D^	0.2	492

BM: Birth-mother. Caregivers choosing the ^S^suspect or ^D^don’t know options were excluded (chi-square or t-test *P<.05, **P<.005, ***P≤.0001).

The majority (76.3%) of adoptive parents were able to confidently provide information about illicit drug exposures including methamphetamine, marijuana, or cocaine. Two-thirds of the birth mothers of Adoptive children relative to only one out of every twenty-five Comparison moms used methamphetamine during pregnancy (OR = 41.0). The biological mothers of Adoptive children more commonly smoked cigarettes (OR = 25.9) and marijuana (OR = 6.2) as well as consumed alcohol (OR = 30.4) during pregnancy. Examination of maternal drug use patterns specific to the third-trimester revealed continued polysubstance use, particularly of nicotine and alcohol (Table 2). Academically, Adoptive children were more likely to be behind Comparison children in school (OR = 5.2) and also behind their peers on math (OR = 3.0) and reading (OR = 3.3).

**Table 2:** Third-trimester drug exposure among adoptive and comparison children in Study I\(BRIEF: Behavioral Rating Inventory of Executive Function) or II (CBCL: Child Behavior Checklist).

	Study I: BRIEF				Study II: CBCL			
	Adoptive	N	Comparison	N	Adoptive	N	Comparison	N
Nicotine	86.1%***	36^SD^	19.1%	378	61.9%	21^SD^	16.8%	489
Alcohol	68.4%***	19^SD^	9.9%	375	33.3%	15^SD^	0.8%	489
Methamphetamine	45.0%***	40^D^	1.6%	378	63.3%***	30^D^	1.2%	492
Marijuana	40.0%***	40^D^	6.3%	378	20.0%**	30^D^	5.5%	492
Cocaine	20.0%***	40^D^	0.8%	378	16.7%***	30^D^	0.4%	492
Methadone	7.5%**	40^D^	0.3%	378	3.3%	30^D^	0.0%	492
Barbiturates	5.0%*	40^D^	0.3%	378	6.7%**	30^D^	0.0%	492
Heroin	5.0%*	40^D^	0.3%	378	3.3%	30^D^	0.0%	492
Oxycontin	2.5%	40^D^	0.0%	378	3.3%	30^D^	0.0%	492

Caregivers choosing the ^S^suspect or ^D^don’t know options were excluded (chi-square *P<.05, **P<.005, ***P<.0005).

### Behavior Rating Inventory of Executive Function (BRIEF)

**Figure 1:**
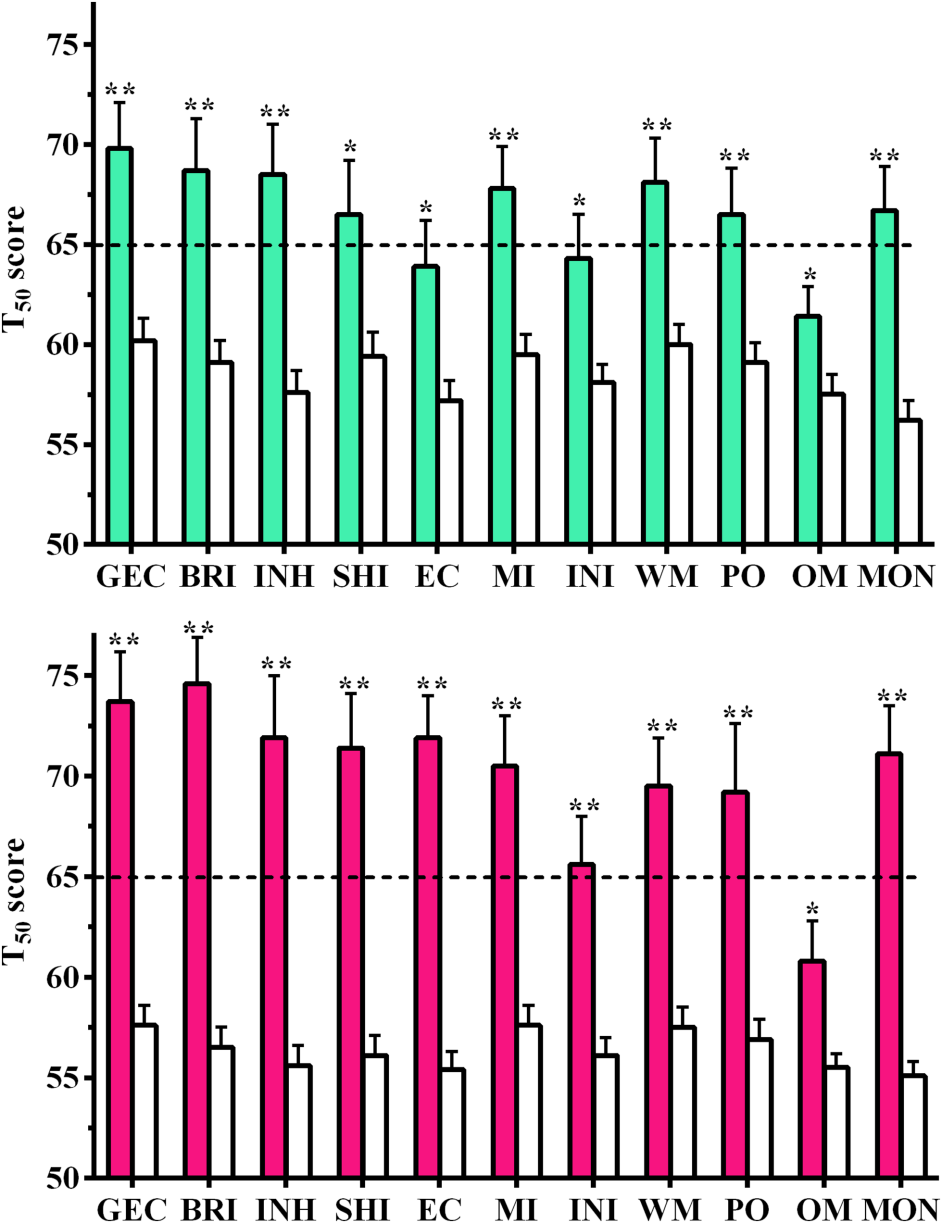
Behavioral Rating Inventory of Executive Function parental ratings of Adoptive (filled bars) and Comparison (open bars) boys (top) and girls (bottom). Global Executive Composite (GEC), Behavioral Regulation Index (BRI), Inhibit (INH), Shift (SHI), Emotional Control (EC), Metacognition Index (MI), Initiate (INI), Working Memory (WM), Plan Organize (PO), Organization of Materials (OM), and Monitor (MON), (*P<.05, **P<.005).

Mean inconsistency ratings did not differ between groups (Adoptive = 3.6±2.1, Comparison = 3.3±1.9) but Adoptive (2.5±2.2) children scored higher than Comparison (1.1±1.8) children on Negativity (*t*(435) = 5.59, *P*<.0005). Similarly, Negativity scores were more likely to be Highly Elevated (≥7) among Adopted (8.5%) than Comparison (1.6%) children (χ^2^(1) = 9.87, *P*<.005). Adopted boys exhibited statistically significant elevations on the total (Global Executive Composite, *d* = .69), both broadband scores (*d = *.64 to .66), and all scales (*d* = .42 to .79). The mean BRIEF scores were in the clinically significant (T_50_≥65) range for Adopted boys on all scales except Organization of Materials (*d* = .42) and Emotional Control (*d* = .49, Figure 1A).

Similarly, Adopted girls demonstrated statistically significantly higher scores than girls living with their biological parents (mother and/or father) on the Global Executive Composite (*d* = 1.25), the broadband scores (Metacognition *d* = .99, Behavioral Regulation Index *d* = 1.45), and the narrowband scales (*d* = .53 to 1.42). Notably, only Organization of Materials (*d* = .53) was below the clinical cut-off (Figure 1B). Although the means were generally higher among Adopted girls relative to Adopted boys, Emotional Control was the only significant sex difference (*t*(55) = 2.45, *P*<.05). Among the Comparison group, boys were rated as having more problems on Organization of Materials (*t*(374) = 2.00, *P*<.05) and Shift (*t*(373) = 2.19, *P*<.05). The same general pattern of group differences between Adoptive and Comparison children was retained when the children with ADHD were excluded (Figure S1A, *d* = .61 to 1.19). There was no evidence that these group differences dissipated with increasing age. In fact, the effect size was larger in adolescents relative to children on ten out of eleven BRIEF scales (Table S1). The percentage of each group that was above the clinical cut-off is shown in Table 3.

**Table 3:** Percentage of Adopted and Comparison boys and girls with clinically significant (T_50_≥65) problems on the Behavioral Rating Inventory of Executive Function.

	Boys			Girls		
	Adoptive (%)	Comparison (%)	Odds Ratio	Adoptive (%)	Comparison (%)	Odds Ratio
Global Executive Composite	72.7*	42.5	3.61	76.0**	27.5	8.34
Behavioral Regulation Index	69.7**	35.5	4.18	80.0**	27.0	10.82
Inhibit	69.7**	32.3	4.83	68.0**	24.3	6.61
Shift	54.5	36.6	2.08	68.0**	26.5	5.91
Emotional Control	51.5*	30.5	2.42	79.2**	22.8	12.90
Metacognition Index	66.7*	38.7	3.17	72.0**	25.9	7.35
Initiate	54.5*	34.9	2.23	56.0*	25.4	3.74
Working Memory	57.6	39.6	2.07	72.0**	28.0	6.60
Plan Organize	60.6*	35.5	2.80	58.3*	28.7	3.47
Organization of Materials	48.5*	29.4	2.26	40.0	22.8	2.26
Monitor	69.7%**	30.6%	5.20	72.0%**	22.8%	8.73

Adopted Boys N = 25; Comparison Boys N = 189; Adopted Girls N = 33; Comparison Girls N = 186. (chi-square *P<.05, or **P<.0005).

### Sample characteristics in Study II

Geographically, three-fifths of participants were from the West-Coast of the United States (36.5% Oregon, 12.9% Washington, 11.6% California). Table 1 shows that the Adoptive (N = 54) and Comparison (N = 495) groups did not differ significantly based on child age, sex, ethnicity, or prematurity but that Adoptive children again exhibited more academic difficulties (OR = 3.0), specifically in being behind peers in math (OR = 2.7) and reading (OR = 2.5). The Adopted children typically began living with their current family before the age of three (Mean = 2.7±0.4, Median = 1.9, Min = 0, Max = 11.7 years). Adopted children were more likely to have a wide variety of psychiatric, neurological, and other medical conditions including Fetal Alcohol Syndrome (OR = 30.0), an Attachment Disorder (OR = 23.4), an Anxiety Disorder (OR = 2.4) specifically Post-Traumatic Stress Disorder (OR = 10.0), a Cognitive Delay (OR = 8.0), Sensory Integration Disorder (OR = 7.8), Tourette Disorder (OR = 6.3), Bipolar Disorder (OR = 5.5), a visual impairment (OR = 3.3), or ADHD (OR = 2.6). The birth mother of Adoptive children had lower incomes and education but family income did not currently differ between Adoptive and Comparison children. Only half of Adoptive respondents were able to provide information about prenatal exposures to illicit drugs (methamphetamine, marijuana, cocaine, Oxycontin) and even fewer could definitively answer items regarding alcohol or nicotine. The majority of Adoptive, relative one fifth or less of Comparison children, had been exposed to alcohol, nicotine or methamphetamine during pregnancy. Prenatal marijuana, cocaine, barbiturates, and Oxycontin exposures were also more common among Adoptive children. Methamphetamine and nicotine exposures were also prevalent in Adopted, but not Comparison, children in the third trimester (Table 2).

### Child Behavior Checklist (CBCL)

**Figure 2:**
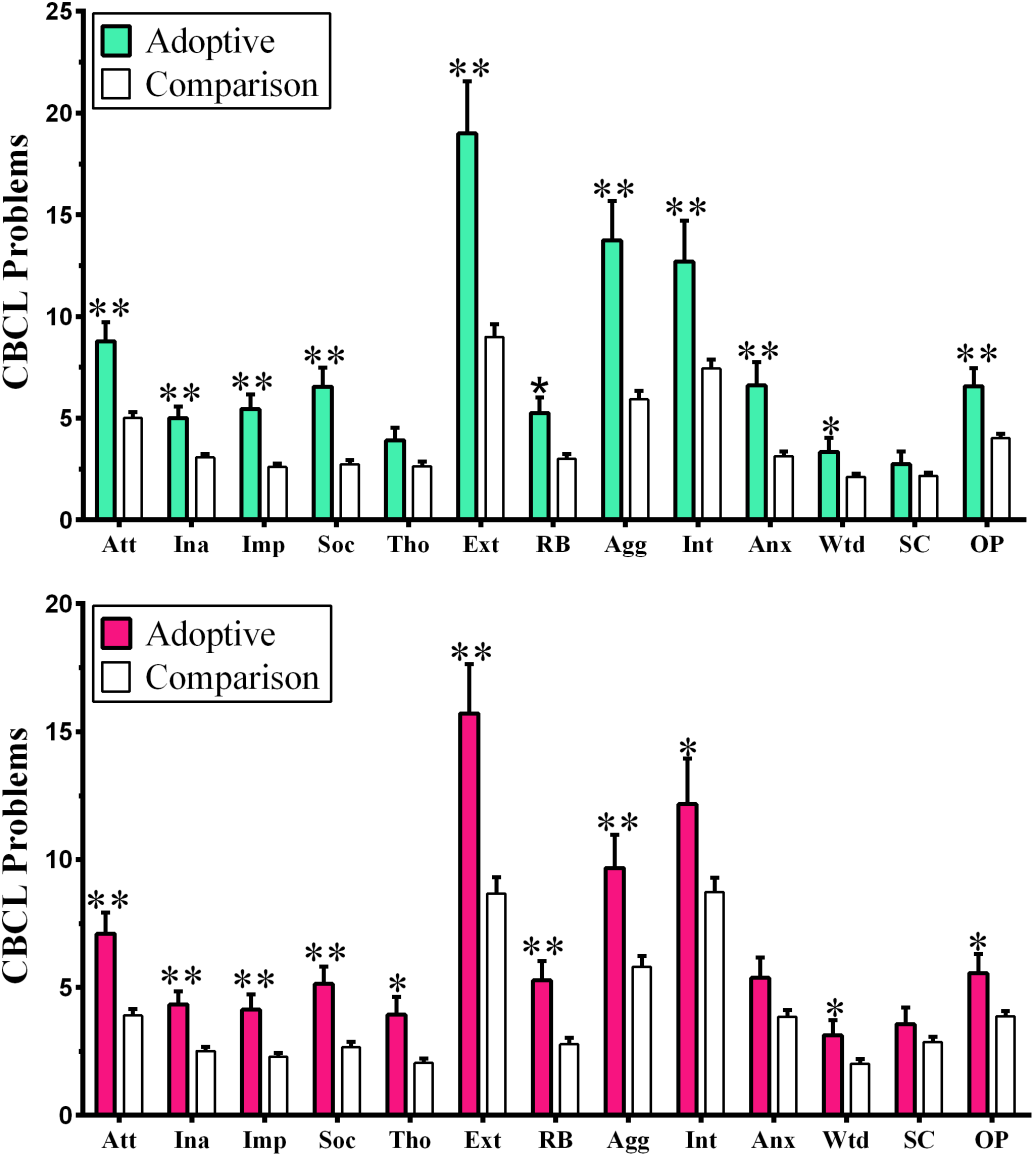
Child Behavior Checklist (CBCL) ratings of Adoptive and Comparison boys (top) and girls (bottom). Attention (Att), Inattention (Ina), Impulsivity (Imp), Social (Soc) Problems, Thought (Tho) Problems, Externalizing (Ext), Rule Breaking (RB), Aggression (Agg), Internalizing (Int), Anxious-Depressed (Anx), Withdrawn-Depressed (Wtd), Somatic Complaints (SC), or Other Problems (OP), (*P<.05, *P<.005).

Adopted boys were rated at having more Attention (*d* = .86), Externalizing (*d* = .91), and Internalizing (*d* = .63) problems than Comparison boys. Other group differences included Aggressive Behavior (*d* = .98), Impulsivity (*d* = .97), Social Problems (*d* = .97), and Anxiety/Depression (*d* = .77, Figure 2A). Adopted girls exhibited a generally similar pattern with more Attention (*d = *.73), Externalizing (*d* = .71), and Internalizing (*d* = .38) problems than Comparison girls (Figure 2B). The same pattern of group differences between Adopted and Comparison children was observed with children with ADHD excluded (Figure S1B, *d* = 0.89 to 1.27). Among Comparison children, boys showed the anticipated increase in Attention Problems (*t*(482) = 2.91, *P*<.005), specifically Inattention Problems (*t*(486) = 2.45, *P*<.05) as well as Thought Problems (*t*(481) = 2.13, *P*<.05) relative to Comparison girls. Conversely, Comparison girls exhibited the expected increase relative to boys for Anxiety and Depression (*t*(486) = −2.77, *P*<.01). However, no sex differences were present among Adopted children. Adopted boys and girls more frequently had clinically significant Attention and Aggression problems but only boys had more Social and Anxiety/Depression problems and only girls had more Thought Problems (Table 4). The presence of clinically significant problems was equally common among children and adolescents (Table S2). Among the Adoptive children, there were no appreciable CBCL differences between children adopted at younger (<1.5) versus older ages (*P*>.11).

**Table 4:** Percentage of Adopted and Comparison boys and girls with clinically significant (T_50_>63) problems on the Child Behavior Checklist.

	Boys			Girls		
	Adoptive (%)	Comparison (%)	Odds Ratio	Adoptive (%)	Comparison (%)	Odds Ratio
Internalizing						
Anxious/Depressed	21.7*	4.4	6.03	13.3	8.8	1.60
Withdrawn/Depressed	17.4	7.6	2.57	12.9	7.9	1.73
Somatic Complaints	8.7	6.4	1.39	13.3	10.8	1.27
Social	30.4*	6.5	6.29	10.0	6.8	1.54
Thought	17.4	9.4	2.04	23.3*	8.4	3.30
Attention	22.7*	7.3	3.74	32.3**	10.1	4.23
Externalizing						
Rule-Breaking	17.4	8.5	2.27	13.8	8.0	1.84
Aggression	39.1*	10.9	5.26	20.0*	7.2	3.24

Adopted Boys N = 22; Comparison Boys N = 247; Adopted Girls N = 33; Comparison Girls N = 186. (chi-square *P<.05, or **P<.0005).

## Discussion

The principal findings of this report are that adoptive children, the majority of whom had a history of prenatal exposure to methamphetamine, nicotine, and alcohol, exhibited a pronounced and diffuse elevation in problems with executive function as well as psychiatric symptomology. Importantly, adoptive children have been included in the samples of several behavioral teratology reports [4], specifically of children exposed to alcohol [18], cocaine [19], and methamphetamine [10], [20]. These reports can be compared with studies of adoptive children without a known history of substance exposure. A meta-analysis of over 100,000 children determined that there were small, but significant, increases in Externalizing (*d* = .24) and Internalizing (*d = *0.16) in adopted, relative to nonadopted children [2]. Interestingly, preschoolers with a history of methamphetamine/nicotine exposure showed a relatively focused behavioral profile with higher (i.e. more problematic) ratings, primarily made by their biological parents, for emotional reactivity (*d* = .16) and anxiety/depression (*d* = .16) but no significant elevations at this age (3 and 5) in Attention or Externalizing problems [20]. There is also a large, albeit contentious, literature reviewed in [15] documenting CBCL elevations in the offspring of women that smoked but used other recreational drugs at low levels. For example, New Zealand toddlers whose mothers smoked cigarettes were more likely to be rated as having clinically significant Somatic (OR = 2.4) and Externalizing (OR = 1.8) problems [21]. Prenatal alcohol, unlike cocaine, has generally been reported to be associated with CBCL abnormalities which are not mediated by the postnatal environment [22]. Importantly, the issue of the threshold alcohol dose necessary to induce CBCL increases has not been conclusively determined but the pattern of alcohol intake is likely a key variable [22]–[24]. The present CBCL ratings, either expressed as the mean or as the percentage above the clinically significant cut-off, were increased in polysubstance exposed adoptive children and is generally consistent with a large body of evidence [22]. Importantly, the magnitude of group differences with effect sizes in the moderate to large size in this sample is indicative a particularly severe pattern of psychopathology.

Polysubstance exposed adoptive children also exhibited statistically and clinically significantly higher (i.e. more problematic) executive function ratings. In contrast to the substantial wealth of prior research with the CBCL, the available information using the BRIEF is much more limited. Importantly, the preschool version of this instrument has been employed to examine internationally adopted children, albeit with an unknown prenatal history, and identified relatively subtle group differences. Only 11% of adoptees from a variety of countries fell into the problem range on the Global Executive Composite [25]. Perhaps unexpectedly, three scales were significantly lower (i.e. less problems) relative to the BRIEF standardization sample among Russian born preschoolers adopted into families in the United States. Further, the BRIEF means were within a half standard deviation (T_50_<55) among school aged children unless they were adopted after age 1.5 in which case the averages were still below the clinical cut-off [26]. The BRIEF profile observed among Adopted and polysubstance exposed children is much more pronounced than that observed among adopted children that did not have an in utero exposure history. Overall, these findings indicate that adoption per se is only responsible for a portion of the variance in BRIEF ratings and that other factors associated with the birth-mother may be responsible. Use of licit and illicit drugs is likely a key factor although we cannot discount the involvement of stress or other sub-optimal aspects of the prenatal environment either acting alone or synergistically with the teratogens.

The large group difference identified between Adopted/polysubstance exposed and comparison children is also of interest when considering findings observed following exposure to other recreational drugs and at different ages. There were significant BRIEF elevations among methamphetamine/alcohol/nicotine exposed children (ages 7 to 9) living with their birth parents which tended to be more severe among adoptive/exposed children [10]. The present results with both the BRIEF and CBCL substantially elaborates upon earlier outcomes [10] and indicates that the atypical profile is not limited to the period shortly after starting school and persists into adolescence. The BRIEF profile, specifically with mean elevations two standard deviations above that of the standardization sample and with the majority of Adopted children meeting the criteria for a clinically significant impairment on all scales with the exception of Organization of Materials, shows striking similarities to that described previously for children diagnosed with Fetal Alcohol Spectrum Disorders [6]. Although only a small subset (10–11%) of our sample included children with Fetal Alcohol Syndrome (FAS), this condition is not trivial to diagnose [27]. Some caregivers are also hesitant to have a formal evaluation given due to concerns of child stigmatization so it is very possible that some children were undiagnosed.

Sex differences are clearly evident in the prevalence of a wide variety of psychiatric conditions including disorders like ADHD which rely on executive function [28]. The question of whether one sex is more vulnerable to prenatal substance exposure has been the focus of substantial empirical attention with some, albeit sporadic, findings [11], [29]. For example, preschool girls, but not boys, exposed throughout pregnancy to methamphetamine and nicotine demonstrated elevated N-acetyl compounds and decreased myoinositol in the frontal white matter [30]. In Study I adopted females showed higher mean ratings than adopted males on Emotional Control but no appreciable sex differences were evident when using clinical cut-offs with the BRIEF. In Study II, both sexes showed an equivalent pattern when the mean raw scores (i.e. not corrected for age and sex) were evaluated but there was some indications of a sex difference when the percentage of children that met clinically significant criteria (i.e. corrected for age and sex) were examined with boys, but not girls, more commonly meeting this criteria for Anxious/Depressed and Social Problems with the CBCL.

There are many ambiguities and uncertainties associated with studies of adopted children and some limitations as well as future directions are noteworthy. One ongoing challenge in determination of the risks associated with recreational drug use during pregnancy, and particularly among children who were subsequently adopted, is the veracity of information about the timing and intensity of substance abuse. Although hair analysis provides a long window of detection (months) and this technology is rapidly advancing for detection of methamphetamine [31], nicotine [32], as well as other drugs [33], collection of this biological matrix was not a common practice when the children in this study were born. Similarly, meconium testing for alcohol metabolites may prove to be the most sensitive index to complement self-reports [34]. Although it may be tempting to speculate that specific agents, especially alcohol, contribute to the neurobehavioral profile observed, inferences of this type should be made with substantial caution given the indirect, and frequently incomplete, nature of the information provided by adoptive mothers. Obtaining medical records of the birth mother without her consent in order to verify the adoptive mother’s reports of prenatal drug exposures is not feasible due to Health Insurance Accountability and Portability Act regulations and was not attempted for this anonymous online investigation. In theory, while longitudinal research which involves a representative sample of biological mothers abusing drugs and the adoptive mothers would extend upon the current findings, a variety of logistical and ethical challenges considerably limits the likelihood of such a hypothetical study being conducted in the immediate future. Atypical CBCL and BRIEF scores among polysubstance exposed adoptive children may also be compared to ratings made by birth parents of children that did, and did not, have a history of recreational drug exposures, particularly to nicotine [8], [15]. While the present dataset is novel and well powered, future research that corroborates and extends upon the maternal reports of psychopathology is needed including teacher reports as well as direct neurobehavioral and neurophysiological assessments of the children. Additional studies are also needed to identify the most optimal postnatal environment and psychoeducational interventions for polysubstance exposed adopted children with the goal of ameliorating deficits in executive function and enhancing mental health.

In conclusion, adoptive children with histories of prenatal exposure to recreational drugs had statistically and substantially greater difficulties with executive (or self-regulatory) and behavioral functioning as assessed by parent ratings relative to a nonadopted comparison group, and the proportion of adopted children with clinically significant psychopathology (i.e., scores>63) was also much greater. The CBCL and BRIEF have been employed previously with adopted children and do not show group differences of the magnitude reported here indicating that other factors are responsible. Adoptive children are not a homogenous group [35] and generalizations based on this descriptive cross-sectional dataset should be limited exclusively to the adoptive offspring of birth mothers that used alcohol and other recreational substances during pregnancy. This report does contribute to a wide body of evidence [3], [4], [6], [22], [36] which supports continued vigilance to minimize the prevalence of children exposed prenatally to alcohol and other recreational drugs during pregnancy.

## Supporting Information

Figure S1 Behavioral Rating Inventory of Executive Function (N = 335, top) and Child Behavior Checklist (N = 450, bottom) ratings with of Adoptive (filled bars) and Comparison (open bars) with ADHD children excluded (* ***P*** <.05, ** ***P*** **<.005).** BRIEF Indexes and Scales: Global Executive Composite (GEC), Behavioral Regulation Index (BRI), Inhibit (INH), Shift (SHI), Emotional Control (EC), Metacognition Index (MI), Initiate (INI), Working Memory (WM), Plan Organize (PO), Organization of Materials (OM), and Monitor (MON). CBCL Indexes and Scales: Attention (Att), Inattention (Ina), Impulsivity (Imp), Social (Soc), Thought (Tho), Externalizing (Ext), Rule Breaking (RB), Aggression (Agg), Internalizing (Int), Anxious-Depressed (Anx), Withdrawn-Depressed (Wtd), Somatic Complaints (SC), or Other Problems (OP).
(TIF)

Table S1 **Behavioral Rating Inventory of Executive Function standardized (T_50_) scores by offspring age and parental relationship.**
(DOCX)

Table S2 **Percentage of Adopted and Comparison children with clinically significant (T_50_>63) problems on the Child Behavior Checklist by offspring age and parental relationship.**
(DOCX)

Data S1 **Raw data from Study I.**
(SYZ)

Data S2 **Raw data from Study II.**
(SYZ)

Materials S1 (PDF)
