# Supplementary material for: Executive Function and Mental Health in Adopted Children with a History of Recreational Drug Exposures
Source: PLoS One. 2014 Oct 22;9(10):e110459. doi: 10.1371/journal.pone.0110459 (PMC4206404; doi:10.1371/journal.pone.0110459)
Supplement: Table S1 — Behavioral Rating Inventory of Executive Function standardized (T50) scores by offspring age and parental relationship. (DOCX) [file pone.0110459.s002.docx]

**Table S1.** Behavioral Rating Inventory of Executive Function standardized (T_50_) scores by offspring age and parental relationship.

* *P* < .05, or ** *P* < .0005.

____________________________________________________________________________________________________________________________ Children Adolescents

Adoptive (N = 44) Comparison (N = 259) Adoptive (N = 14) Comparison (N = 116)

Mean (SD) Mean (SD) Cohen’s *d* Mean (SEM) Mean (SEM) Cohen’s *d*

Global Executive Composite 70.5 (13.1)** 58.2 (13.8) .92 74.5 (12.7)** 60.6 (14.1) 1.04

Behavioral Regulation Index 70.2 (13.6)** 57.4 (14.1) .92 74.5 (14.3)** 58.6 (15.1) 1.08

Inhibit 68.8 (14.1)** 56.6 (13.6) .89 73.4 (16.8)** 56.7 (15.1) 1.05

Shift 68.4 (15.2)** 56.8 (14.4) .64 69.4 (14.1)* 59.7 (15.7) .66

Emotional Control 66.3 (13.2)** 56.2 (13.4) .77 70.1 (11.2)** 56.6 (13.8) 1.08

Metacognition Index 68.1 (12.4)** 57.6 (13.2) .82 71.8 (11.7)** 60.7 (13.4) .88

Initiate 63.8 (12.3)** 56.4 (12.3) .60 68.2 (12.2)* 58.5 (12.9) .77

Working Memory 68.2 (12.1)** 57.7 (12.9) .84 70.5 (13.8)* 61.0 (15.2) .66

Plan Organize 66.9 (15.0)** 57.2 (13.8) .67 70.0 (13.4)* 59.8 (12.9) .78

Organization of Materials 60.9 (9.5)** 56.1 (10.3) .49 61.9 (8.1) 57.4 (9.4) .51

Monitor 67.3 (12.6)** 54.7 (13.6) .96 72.6 (12.0)** 57.9 (13.5) 1.16

____________________________________________________________________________________________________________________________
