# Supplementary material for: Executive Function and Mental Health in Adopted Children with a History of Recreational Drug Exposures
Source: PLoS One. 2014 Oct 22;9(10):e110459. doi: 10.1371/journal.pone.0110459 (PMC4206404; doi:10.1371/journal.pone.0110459)
Supplement: Table S2 — Percentage of Adopted and Comparison children with clinically significant (T50>63) problems on the Child Behavior Checklist by offspring age and parental relationship. (DOCX) [file pone.0110459.s003.docx]

**Table S2.** Percentage of Adopted and Comparison children with clinically significant (T_50_ > 63) problems on the Child Behavior Checklist by

offspring age and parental relationship. * *P* < .05, or ** *P* < .0005.

_________________________________________________________________________________________________________________________________

Children Adolescents

Adoptive Comparison Adoptive Comparison

N = 39 N = 310 N = 14 N = 174

% % Odds Ratio % % Odds Ratio

Attention 28.2** 9.0 4.0 28.6* 8.0 4.6

Social 15.4 6.8 2.5 28.6* 6.4 5.9

Thought 20.5* 8.9 2.6 21.4 8.8 2.8

Externalizing

Rule-Breaking 15.8 7.1 2.5 14.3 10.4 1.4

Aggression 28.9** 9.3 4.0 26.7* 8.6 3.9

Internalizing

Anxious/Depressed 15.4* 6.3 2.7 21.4 6.9 3.7

Withdrawn/Depressed 10.3 6.6 1.6 26.7 9.7 3.4

Somatic Complaints 10.3 5.7 1.9 14.3 13.8 1.0

__________________________________________________________________________________________________________________________________
