# Supplementary material for: Executive Function and Mental Health in Adopted Children with a History of Recreational Drug Exposures
Source: PLoS One. 2014 Oct 22;9(10):e110459. doi: 10.1371/journal.pone.0110459 (PMC4206404; doi:10.1371/journal.pone.0110459)
Supplement: Materials S1 — (PDF) [file pone.0110459.s006.pdf]

Child Behavior (OCTRI 10933)

You are being asked to participate in a project conducted through the Psychology Department at Northern Arizona University by Melissa Birkett that involves research. The researcher is required to receive your informed consent before you participate in this project.

This project has been reviewed and approved by the Northern Arizona University Institutional Review Board (IRB) for the Protection of Human Subjects in Research. Contact the Human Research Protections Office at 928-523-4236 if you have any questions about (1) the conduct of the project, (2) your rights as a research participant, or (3) a research-related injury. Any other questions about the research project should be directed to: Melissa Birkett, Ph.D. (Melissa.Birkett@nau.edu).

Your participation in research is voluntary. If you refuse to participate, there are no penalties or loss of benefits or services that you are otherwise entitled to. If you decide to participate and then withdraw or skip a question there are no penalties or loss of benefits or services. Whether or not you choose to participate in this project will have no effect on your relationship with NAU now or in the future.

A basic explanation of the project is written below. After you have read this information, please indicate your consent at the bottom of this page.

PROJECT PURPOSE:

The purpose of this on-line survey is to gather information about prenatal environment, possible exposure to drugs prenatally, and your child’s behavior. Parents whose children WERE and WERE NOT exposed to drugs prenatally are being asked to participate in this study.

EXPLANATION OF PROCEDURES:

This study will involve answering questions from an established questionnaire; the Child Behavior Checklist (CBCL).

CONFIDENTIALITY:

No identifying information will be collected during this research. Information from this research will not be released to anyone outside of this research project. Your responses will not be discussed with anyone not involved in the research. Only researchers associated with the project will have access to this information.   Any information from this study to be presented or published, will be in aggregate form only and individuals will not be identified.

COMPENSATION:

If you participate in this research for extra credit through the Sona system, you will receive 0.5 Sona research credits. No other compensation will be awarded.

BENEFITS:

The overall benefits of this research include gaining a better understanding of the relationship between prenatal environment and behavior of children.

RISKS:

This study involves minimal risk. Risks in this study are not anticipated to be any greater than those involved in day-to-day activities (working with a computer, answering questions, reading etc.).

CONSENT:

I have read the above information about this study and have been given an opportunity to ask questions (via email).

Do you consent to be part of this research?

☐ Yes
☐ No

Please enter the current date (ex. mm-dd-yyyy) ________________

What country do you live in? ________________________________

What state/province do you live in? ________________________________

What is your relation to the child?

☐ Adoptive parent
☐ Foster parent
☐ Biological mother
☐ Biological father
☐ Other family member
☐ Other

Please describe your relationship to the child

For what % of the child's life have they lived with you? ____________________

What is the biological sex of your child?

- ☐ Male
- ☐ Female

What is the ethnicity of your child?

- ☐ White
- ☐ Black or African American
- ☐ Asian
- ☐ Hispanic or Latino
- ☐ Native Hawaiian
- ☐ Pacific Islander
- ☐ American Indian
- ☐ Alaska Native
- ☐ Other

Please specify the ethnicity of your child

When was your child born? (ex. mm-dd-yyyy) ____________

Was your child born premature?

- ☐ Yes
- ☐ No
- ☐ I don't know

How many weeks premature was your child born? ____________________

After your child was born, was he/she put in an intensive care unit?

- ☐ Yes
- ☐ No
- ☐ I don't know

How old was the biological mother when she became pregnant? ____________________

What is the ethnicity of the biological mother?

- ☐ White
- ☐ Black or African American
- ☐ Asian
- ☐ Hispanic or Latino
- ☐ Native Hawaiian
- ☐ Pacific Islander
- ☐ American Indian
- ☐ Alaska Native
- ☐ Other

Please specify the ethnicity of the biological mother ______________________________

Did the biological mother engage in physical activities or exercise during her pregnancy?

- ☐ Yes
- ☐ No
- ☐ I don't know

On average, how many hours a week did the biological mother engage in physical activity or exercise during her pregnancy? ______________________________

What is the highest grade of schooling that the biological mother has completed?

- ☐ Jr High/High school grade 7
- ☐ Jr High/High school grade 8
- ☐ Jr High/High school grade 9
- ☐ Jr High/High school grade 10
- ☐ Jr High/High school grade 11
- ☐ Jr High/High school grade 12
- ☐ GED
- ☐ College grade 13
- ☐ College grade 14
- ☐ College grade 15
- ☐ College grade 16
- ☐ Graduate school 17
- ☐ Graduate school 18
- ☐ Graduate school 19
- ☐ Graduate school 20+

What was the total family income (before taxes) of the biological mother when she became pregnant? Include the biological mother's income, her husband's or partner's income, and any other income she may have received

- ☐ Less than $10,000
- ☐ $10,000 to $14,999
- ☐ $15,000 to $19,999
- ☐ $20,000 to $24,999
- ☐ $25,000 to $34,999
- ☐ $35,000 to $49,999
- ☐ $50,000 or more
- ☐ Prefer not to disclose or unknown

What is the total family income (before taxes) in the home that the child is currently living?

- ☐ Less than $10,000
- ☐ $10,000 to $14,999
- ☐ $15,000 to $19,999
- ☐ $20,000 to $24,999
- ☐ $25,000 to $34,999
- ☐ $35,000 to $49,999
- ☐ $50,000 or more
- ☐ Prefer not to disclose or unknown

Has the child ever lived in foster care?

- ☐ Yes
- ☐ No

With whom is the child currently living? Please check all that apply

- ☐ With the biological mother
- ☐ With the biological father
- ☐ With other members of the family
- ☐ With adoptive parents
- ☐ In Foster care
- ☐ Other

Please specify with whom the child is currently living ______________________________

The following questions will ask about your child and the biological mother's development and psychological health

What type of school is your child attending?

- ☐ Public school
- ☐ Home school
- ☐ Private school
- ☐ Alternative education program
- ☐ Other

Please specify what type of school is your child attending?

Your child's performance at school is

- ☐ Below age level
- ☐ At age level
- ☐ Above age level

Please rate your child's performance in math with relation to their scores on the state's/province's standardized tests

- ☐ One grade level behind
- ☐ Slightly below grade level
- ☐ At grade level
- ☐ Slightly above grade level
- ☐ One grade level ahead
- ☐ Not applicable

Please rate your child's performance in reading and writing with relation to their scores on the state's/province's standardized tests

- ☐ One grade level behind
- ☐ Slightly below grade level
- ☐ At grade level
- ☐ Slightly above grade level
- ☐ One grade level ahead
- ☐ Not applicable

Has your child ever been diagnosed with any of the following? Please check all that apply

- ☐ Anxiety
- ☐ Attachment Disorder
- ☐ Attention Deficit Hyperactive Disorder (ADHD)
- ☐ Attention Deficit Disorder (ADD)
- ☐ Autism Spectrum Disorder
- ☐ Aspergers Syndrome
- ☐ Bipolar
- ☐ Brain Trauma
- ☐ Cognitively Delayed
- ☐ Cerebral Palsy
- ☐ Congenital Abnormalities
- ☐ Depression
- ☐ Developmental Disorder
- ☐ Diabetes
- ☐ Dyslexia
- ☐ Epilepsy
- ☐ Fetal Alcohol Syndrome
- ☐ Hearing Impaired
- ☐ Increased Muscle Tone
- ☐ Motor Developmental Delay
- ☐ Physical birth defect
- ☐ Post Traumatic Stress Disorder
- ☐ Schizophrenia
- ☐ Sensory Integration Disorder
- ☐ Short Stature
- ☐ Speech Delay
- ☐ Tourette Syndrome
- ☐ Visually Impaired
- ☐ Other Behavior/ Emotional Disorder
- ☐ Other Genetic Condition
- ☐ Other Neurological Condition
- ☐ Other Psychiatric Condition
- ☐ Other

Please specify your child's diagnosis

Is your child taking any prescription medications?

- ☐ Yes
- ☐ No

Please list each prescription medication that your child is currently taking and the conditions they are used to treat

Has your child taken any additional prescription medications in the past year?

- ☐ Yes
- ☐ No

Please list each prescription medication that your child has taken in the past year and the conditions they are used to treat

Do you suspect that your child may be experimenting with any of the following drugs? Please check all that apply

- ☐ Alcohol
- ☐ Nicotine
- ☐ Marijuana
- ☐ Other
- ☐ None
- ☐ Do not know
- ☐ Prefer not to disclose

Please specify the drugs you suspect your child may be experimenting with

Has the biological mother ever been diagnosed with any of the following? Please check all that apply

- ☐ Anxiety
- ☐ Attachment Disorder
- ☐ Attention Deficit Hyperactive Disorder
- ☐ Attention Deficit Disorder
- ☐ Autism Spectrum Disorder
- ☐ Aspergers Syndrome
- ☐ Bipolar
- ☐ Brain Trauma
- ☐ Cognitively Delayed
- ☐ Cerebral Palsy
- ☐ Congenital Abnormalities
- ☐ Depression
- ☐ Developmental Disorder
- ☐ Diabetes
- ☐ Dyslexia
- ☐ Epilepsy
- ☐ Fetal Alcohol Syndrome
- ☐ Hearing Impaired
- ☐ Increased Muscle Tone
- ☐ Motor Developmental Delay
- ☐ Physical birth defect
- ☐ Post Traumatic Stress Disorder
- ☐ Schizophrenia
- ☐ Sensory Integration Disorder
- ☐ Short Stature
- ☐ Speech Delay
- ☐ Tourette Syndrome
- ☐ Visually Impaired
- ☐ Other Behavior/ Emotional Disorder
- ☐ Other Genetic Condition
- ☐ Other Neurological Condition
- ☐ Other Psychiatric Condition
- ☐ Other
- ☐ Do not Know
- ☐ Prefer not to disclose

Please specify the biological mother's diagnosis

The following questions will ask about the biological mother's prenatal health. Your willingness to be honest is greatly appreciated

Did the biological mother take any prescriptions or over the counter drugs either in the three months before pregnancy or during this pregnancy? Please check all that apply

- ☐ Prenatal supplements
- ☐ Over the counter medication
- ☐ Prescription medication
- ☐ I don't know

Please list all known prescription and/or over the counter medications taken by the biological mother either during the three months before pregnancy or during this pregnancy

Did the biological mother smoke cigarettes during the three months before this pregnancy?

- ☐ Yes
- ☐ No
- ☐ Suspect
- ☐ I don't know

During the three months before this pregnancy, how many days per month did the biological mother smoke cigarettes? ______________________

During the three months before this pregnancy, how many cigarettes (on average) did the biological mother smoke each day? ______________________

Did the biological mother smoke cigarettes during this pregnancy?

- ☐ Yes
- ☐ No
- ☐ Suspect
- ☐ I don't know

During this pregnancy, how many days per month did the biological mother smoke cigarettes? ______________________

During this pregnancy, how many cigarettes did the biological mother smoke each day? ______________________

Did the biological mother smoke cigarettes during the third trimester (weeks 27-42) of this pregnancy?

- ☐ Yes
- ☐ No
- ☐ Suspect
- ☐ I don't know

On average, how many cigarettes per day did the biological mother smoke during the third trimester? ______________________

During the three months before this pregnancy, did the biological mother consume any alcohol (wine, beer, liquor)?

- ☐ Yes
- ☐ No
- ☐ Suspect
- ☐ I don't know

During the three months before this pregnancy, did the biological mother drink 5 or more drinks on any occasion?

- ☐ Yes
- ☐ No
- ☐ Suspect
- ☐ I don't know

During the three months before this pregnancy, how many times per month did the biological mother drink 5 or more drinks on an occasion?

____

During the three months before this pregnancy, how many times per month did the biological mother drink 1-2 drinks on an occasion?

____

During the three months before this pregnancy, did the biological mother drink alcohol almost every day, even if only a small amount?

- ☐ Yes
- ☐ No
- ☐ Suspect
- ☐ I don't know

During this pregnancy, did the biological mother consume any alcohol (wine, beer, liquor)?

- ☐ Yes
- ☐ No
- ☐ Suspect
- ☐ I don't know

During this pregnancy, did the biological mother drink 5 or more drinks on any occasion?

- ☐ Yes
- ☐ No
- ☐ Suspect
- ☐ I don't know

During this pregnancy, how many times per month did the biological mother drink 5 or more drinks on an occasion?

____

During this pregnancy, how many times per month did the biological mother drink 1-2 drinks on an occasion?

____

During the third trimester of this pregnancy (weeks 27-42) did the biological mother consume any alcohol (wine, beer, liquor)?

- ☐ Yes
- ☐ No
- ☐ Suspect
- ☐ I don't know

On average, how many drinks per month did the mother consume during the third trimester?

____

During this pregnancy, did the biological mother drink alcohol almost every day, even if only a small amount?

- ☐ Yes
- ☐ No
- ☐ Suspect
- ☐ I don't know

Has the biological mother ever tried or used Intravenous (IV) street drugs (shot up)?

- ☐ Yes
- ☐ No
- ☐ Suspect
- ☐ I don't know

In general, how many days per month has the biological mother tried or used intravenous (IV) street drugs (shot up)?

____

Has the biological mother ever tried or used cocaine/crack?

- ☐ Yes
- ☐ No
- ☐ Suspect
- ☐ I don't know

In general, how many days per month has the biological mother tried or used cocaine?

____

Has the biological mother ever tried or used methamphetamine?

- ☐ Yes
- ☐ No
- ☐ Suspect
- ☐ I don't know

In general, how many days per month has the biological mother tried or used methamphetamine?

____

Did the biological mother use any of the following drugs during the three months before this pregnancy? Please select all that apply

- ☐ Barbiturates (downers, reds, phenobarb, pentobarb, seconal)
- ☐ Cocaine (crack, rock, coke, soup, powder)
- ☐ Heroin (smack, horse, china white, dove, brown, tar)
- ☐ Inhalants
- ☐ Marijuana (weed, pot)
- ☐ Methadone
- ☐ Methamphetamine (crystal)
- ☐ Oxycontin
- ☐ Other street drugs (acid, PCP, angel dust, MDMA, Ecstacy)
- ☐ None
- ☐ Don't know

Please list the street drugs that the biological mother used during the three months before this pregnancy

Did the biological mother use any of the following drugs during this pregnancy? Please check all that apply

- ☐ Barbiturates (downers, reds, phenobarb, pentobarb, seconal)
- ☐ Cocaine (crack, rock, coke, soup, powder)
- ☐ Heroin (smack, horse, china white, dove, brown, tar)
- ☐ Inhalants
- ☐ Marijuana (weed, pot)
- ☐ Methadone
- ☐ Methamphetamine (crystal)
- ☐ Oxycontin
- ☐ Other street drugs (acid, PCP, angel dust, MDMA, Ecstacy)
- ☐ None
- ☐ Don't know

Please list the street drugs that the biological mother used during this pregnancy

Did the biological mother use any of the following drugs during the third trimester of this pregnancy? Please check all that apply

- ☐ Barbiturates (downers, reds, phenobarb, pentobarb, seconal)
- ☐ Cocaine (crack, rock, coke, soup, powder)
- ☐ Heroin (smack, horse, china white, dove, brown, tar)
- ☐ Inhalants
- ☐ Marijuana (weed, pot)
- ☐ Methadone
- ☐ Methamphetamine (crystal)
- ☐ Oxycontin
- ☐ Other street drugs (acid, PCP, angel dust, MDMA, Ecstacy)
- ☐ None
- ☐ Don't know

Please list the street drugs that the biological mother used during the third trimester of this pregnancy

Please feel free to provide any additional information that you think may be useful

The next section is about your child's behavior. There are 113 items. You are about half way done with this survey!
